# Molecular characterization of sarcomatoid clear cell renal cell carcinoma unveils new candidate oncogenic drivers

**DOI:** 10.1038/s41598-020-57534-5

**Published:** 2020-01-20

**Authors:** Gabriel G. Malouf, Ronan Flippot, Yiyu Dong, Renzo G. Dinatale, Ying-Bei Chen, Xiaoping Su, Eva Compérat, Morgan Rouprêt, Roy Mano, Kyle A. Blum, Hui Yao, Roger Mouawad, Jean-Philippe Spano, David Khayat, Jose A. Karam, Thai H. Ho, Satish K. Tickoo, Paul Russo, James J. Hsieh, Nizar M. Tannir, Abraham A. Hakimi

**Affiliations:** 1 Department of Medical Oncology, Hôpital Pitié Salpêtrière, APHP, Sorbonne Université, Paris, France; 2 Department of Hematology and Oncology, Centre Hospitalier Universitaire Régional de Strasbourg, Institut de Cancérologie de Strasbourg, Strasbourg, France; 3 Department of Cancer and Functional Genomics, Institute of Genetics and Molecular and Cellular Biology, Illkirch, France; 4 Department of Urology, Memorial Sloan Kettering Cancer Center, New York, NY USA; 5 Department of Pathology, Memorial Sloan Kettering Cancer Center, New York, NY USA; 6 Department of Biostatistics and Computational Biology, The University of Texas MD Anderson Cancer Center, Houston, TX USA; 7 Department of Pathology, Hôpital Tenon, APHP, Sorbonne Université, Paris, France; 8 Department of Urology, Hôpital Pitié Salpêtrière, APHP, Sorbonne Université, Paris, France; 9 Inserm UMRS 1136, Sorbonne Université, Paris, France; 10 Department of Urology, MD Anderson Cancer Center, Houston, TX USA; 11 Division of Hematology and Medical Oncology, Mayo Clinic, Scottsdale, AZ USA; 12 Center for Individualized Medicine, Epigenomics Group, Mayo Clinic, Rochester, MN USA; 13 Molecular Oncology, Department of Medicine, Siteman Cancer Center, Washington University, St. Louis, MO USA; 14 Department of Medical Oncology, The University of Texas MD Anderson Cancer Center, Houston, TX USA

**Keywords:** Cancer genetics, Cancer

## Abstract

Sarcomatoid clear-cell renal cell carcinomas (sRCC) are associated with dismal prognosis. Genomic alterations associated with sarcomatoid dedifferentiation are poorly characterized. We sought to define the genomic landscape of sRCC and uncover potentially actionable therapeutic targets. We assessed the genomic landscape of sRCC using targeted panel sequencing including patients with microdissected sarcomatoid and epithelial components. Along with common genomic alterations associated with clear-cell histology, we found that Hippo was one of the most frequently altered pathways in these tumours. Hippo alterations were differentially enriched in sRCC compared to non-sRCC. Functional analysis showed that Hippo members mutations were associated with higher nuclear accumulation of YAP/TAZ, core effectors of the Hippo pathway. In a NF2-mutant sRCC model, *YAP*1 knockdown and *NF2* reconstitution suppressed cell proliferation, tumour growth and invasion, both *in vitro* and *in vivo*. Overall, we show that Hippo pathway alterations are a feature of sRCC, and enable the exploration of the Hippo pathway as a novel potential therapeutic target.

## Introduction

Sarcomatoid dedifferentiation has been reported in ~5% of clear-cell renal cell carcinomas (ccRCC) and is considered one of the most aggressive features of the disease^1^. Sarcomatoid ccRCCs (sRCC) have been associated with high tumour burden and frequent metastatic dissemination, with a synchronous metastasis rate at diagnosis of 70%, compared to 30% in non-sRCCs^2^. Patient prognosis is dismal, with median overall survival duration less than 12 months in patients treated with targeted molecular therapies^3^.

The mechanism underlying sarcomatoid dedifferentiation in ccRCC remains poorly defined. Several recent studies tried to define the genomic landscape of these tumours, with discordant results. Molecular profiling of 26 sarcomatoid renal cell carcinomas with mixed clear cell and non–clear cell histological subtypes identified recurrent alterations of *TP53*, Hippo pathway member *NF2* and cell cycle gene *CDKN2A*^4^. An analysis of another 65 sarcomatoid kidney tumours of varying histological subtypes^5^ reported increased mutations of *PTEN* and chromatin remodelling genes *BAP1* and *SETD2* in clear cell subtypes, increased *NF2* mutations in papillary subtypes and increased *TP53* alterations in both. In one study that performed systematic microdissection of epithelial and sarcomatoid components of renal cell carcinomas^6^, 21 tumours with mixed histology had sarcomatoid components harbouring a greater mutational load than paired epithelial components, as well as greater numbers of mutations of *TP53*, chromatin modifiers *BAP1* and *ARID1* and Hippo pathway gene *FAT2*. Overall, these studies displayed different pictures of sarcomatoid kidney cancers, and key events leading to sarcomatoid dedifferentiation remain unsettled.

Thus, we sought to understand the molecular events associated with sarcomatoid dedifferentiation in ccRCC, looking for alterations that might affect the therapeutic management of these tumours. We show that alterations in the Hippo pathway are frequent in sRCCs, and that inhibition of downstream Hippo effectors inhibit cell proliferation, tumour growth and invasion. Ours is the first report of Hippo pathway alterations as a candidate actionable driver of sarcomatoid dedifferentiation in ccRCC.

## Results

### Targeted sequencing of sRCCs uncovers recurrent genomic alterations

**Figure 1:**
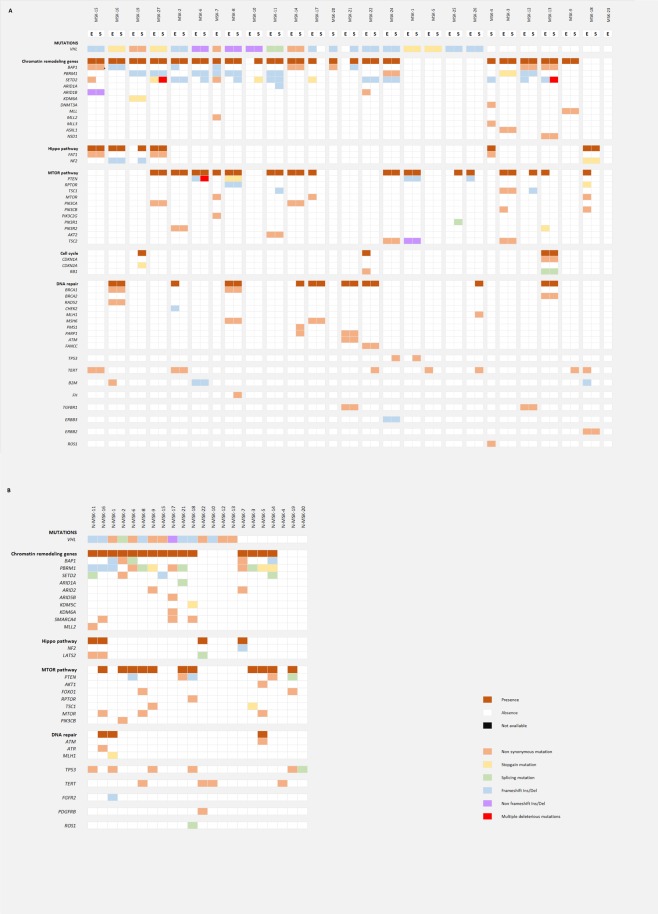
Genomic alterations in sRCCs by targeted panel sequencing (**A**) Genomic alterations identified by targeted sequencing in microdissected sRCCs (N = 27). Epithelial component is labeled E, mesenchymal (sarcomatoid) component is labeled S. (**B**) Genomic alterations identified by targeted sequencing in non-microdissected sRCCs (N = 22).

**Figure 2:**
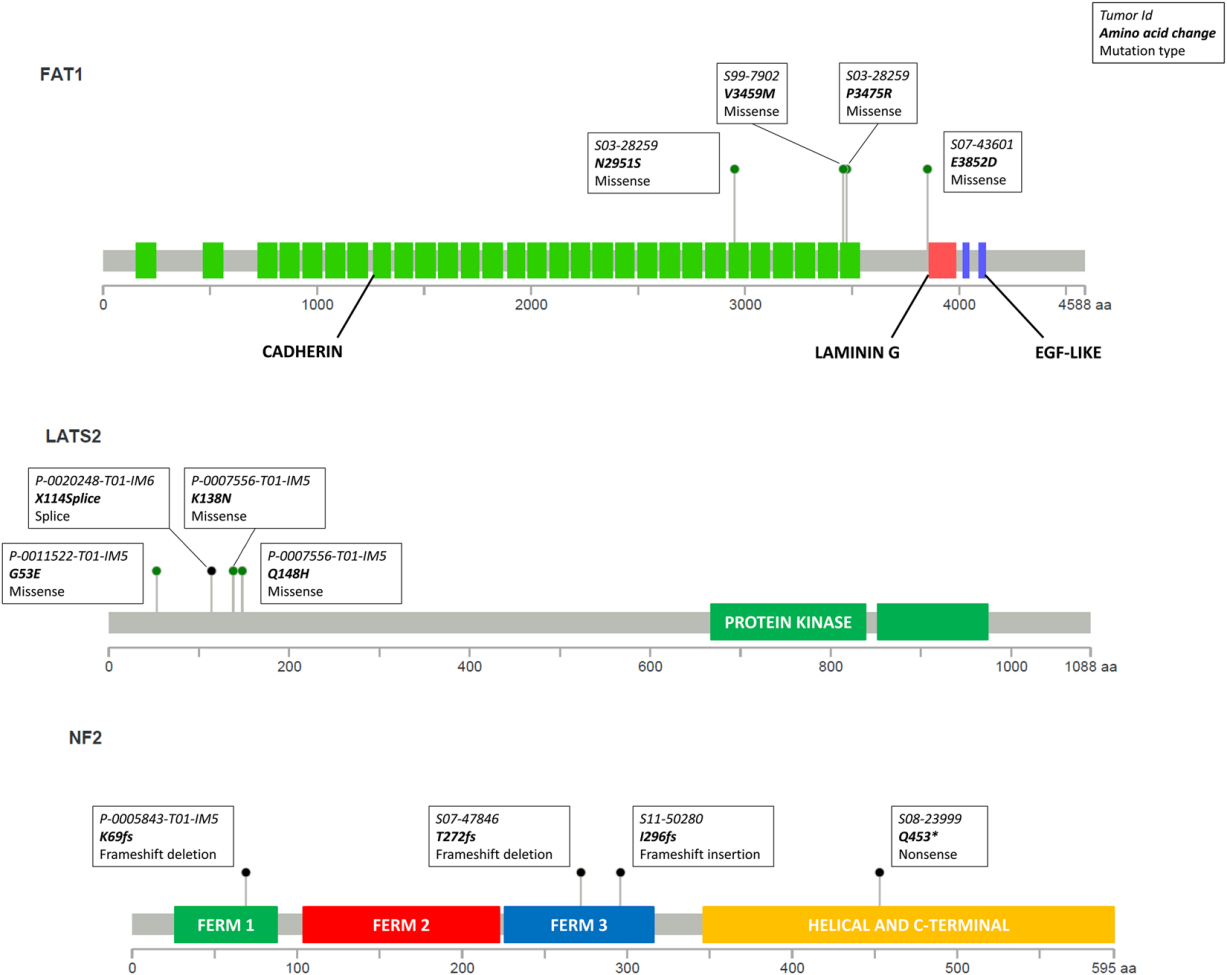
Mapping of Hippo protein alterations in sRCCs.

We first performed targeted sequencing in 50 samples from 27 sRCCs that had been microdissected (Fig. 1A and Supplementary Table S1). Paired sequencing of mesenchymal and epithelial components was available for 23 of these tumours, while 2 had only a mesenchymal component and 2 only an epithelial component available. Across all samples, recurrent oncogenic alterations involved key tumour suppressor genes in ccRCC, including *VHL* (36/50, 72%) and chromatin remodelling genes *SETD2* (20/50, 40%), *PBRM1* (17/50, 34%) and *BAP1* (13/50, 26%). Interestingly, 9 of the 20 mutations of *SETD2* involved a hotspot frameshift mutation (K2fs). Recurrent mutations of *TERT* were identified in 9 samples (18%), including 6 C228T hotspot mutations and 2 C250T hotspot mutations, both located in the *TERT* promoter^7,8^. Additional mutations found in >10% of samples involved MTOR pathway members *PTEN* (7/50, 14%) and *TSC2* (6/50, 12%), as well as Hippo pathway members *NF2* (5/50, 10%) and *FAT1* (5/50, 10%). As recurrent mutations of the Hippo pathway were not previously described with this high rate in ccRCC, we assessed the functional impact of these mutations and found that all mutations of *NF2* and *FAT1* were deleterious and affected functional domains of the proteins (Fig. 2 and Supplementary Table S2).

We then analyzed mutated genes in consideration with putative oncogenic mechanisms in these samples (Fig. 1A). Along with *VHL* mutations, the most frequent alterations affected chromatin remodelling genes (36/50, 72%). In addition to recurrent mutations of *BAP1*, *PBRM1* and *SETD2* previously described, we found mutations of SWI/SNF members *ARID1B* in 3 samples and *ARID1A* in one, as well as mutations of epigenetic regulators *KDM6A* (4%), *MLL (4%)*, *ASXL1 (4%)*, *NSD1* (4%), *DNMT3A (2%)*, *MLL2 (2%)* and *MLL3 (2%)*. Notably, 2 paired epithelial and mesenchymal samples from one tumour presented with convergent mutations of *SETD2* and *NSD1*, which have been described to be associated with an aggressive phenotype in ccRCC^9^. Other frequent alterations involved the MTOR pathway (25/50, 50%), DNA repair (15/50, 30%) and the Hippo pathway (10/50, 20%).

**Figure 3:**
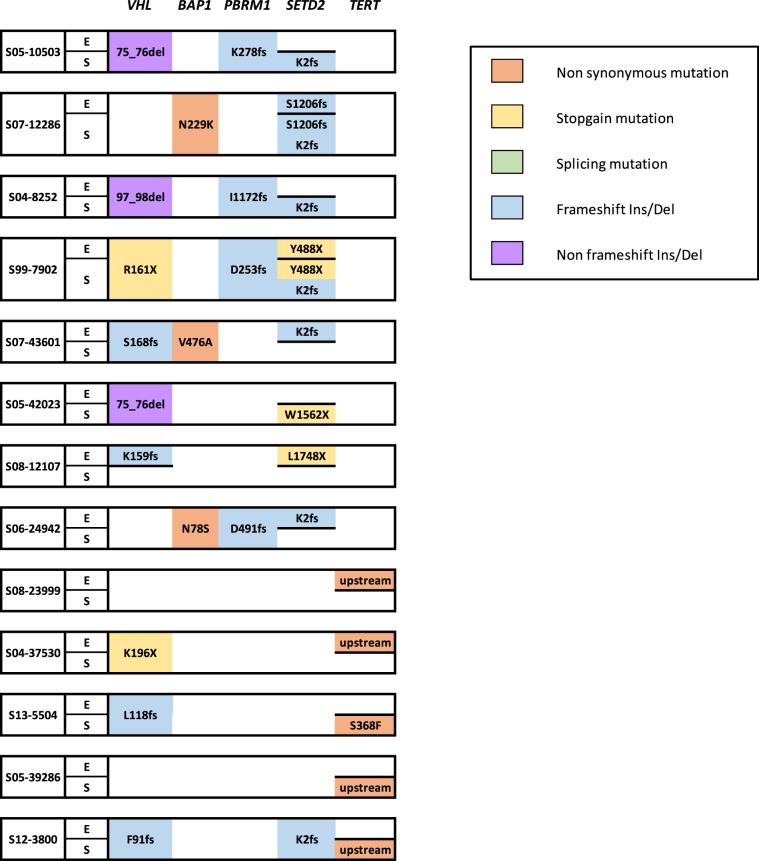
Differential alterations of *SETD2* and *TERT* in epithelial and mesenchymal components of sRCCs.

We then compared the genomic profiles of the 23-paired epithelial and mesenchymal components (Fig. 1A and Supplementary Table S1). Alterations of *SETD2* and *TERT* repeatedly differed between mesenchymal and epithelial components, with 8/20 mutations of *SETD2 and* 5/9 mutations of *TERT* not shared (Fig. 3). These observations are in line with recent studies reporting that *SETD2* alterations are associated with high rates of subclonality^10,11^. Overall, 16 of 23 sRCCs had at least one putative oncogenic mutation specifically found in the mesenchymal component (Fig. 1A). Apart from known ccRCC oncogenic alterations, one tumour harboured *NF2* and *CDKN2A* mutations exclusively in its mesenchymal component, and 2 tumours had *TP53* mutations that were present exclusively in the mesenchymal component of these tumours.

In 22 additional sRCCs, targeted sequencing was performed without prior microdissection (Fig. 1B and Supplementary Table S1). The genomic profiles of these additional samples were concordant with previous findings, with key oncogenic alterations of *VHL* in 68%, of chromatin remodelling genes in 73% and of the MTOR pathway in 50%. In addition, *TP53* alterations were reported in 27%. *TERT* mutations and DNA repair pathway alterations were reported in 18% and 14%, respectively, of these tumours. Interestingly, we again found frequent Hippo pathway alterations (18%). Notably, 3 tumours harboured deleterious mutations of the core Hippo pathway member *LATS2*, one tumour with a splicing mutation and 2 with missense mutations. One additional tumour had a frameshift mutation of *NF2* (Fig. 2).

Overall, in both microdissected and non-microdissected tumours, 10 of the 49 sRCCs displayed deleterious Hippo pathway alterations (20%) in at least one tumour section. We then investigated whether Hippo pathway alterations were more frequent in sRCCs than in the 268 non-sRCCs. The non-sRCCs were predominantly higher risk tumours; only 14 of the 268 (5%) had Hippo pathway alterations, involving *NF2* (6/268), *FAT1* (3/268), *LATS1* (2/268), *LATS2* (3/268) and *YAP1* (1/268). Thus, the frequency of Hippo pathway mutations was significantly higher in sRCCs than in non-sRCCs (p = 0.001).

### YAP/TAZ is upregulated in Hippo-mutant sRCCs

**Figure 4:**
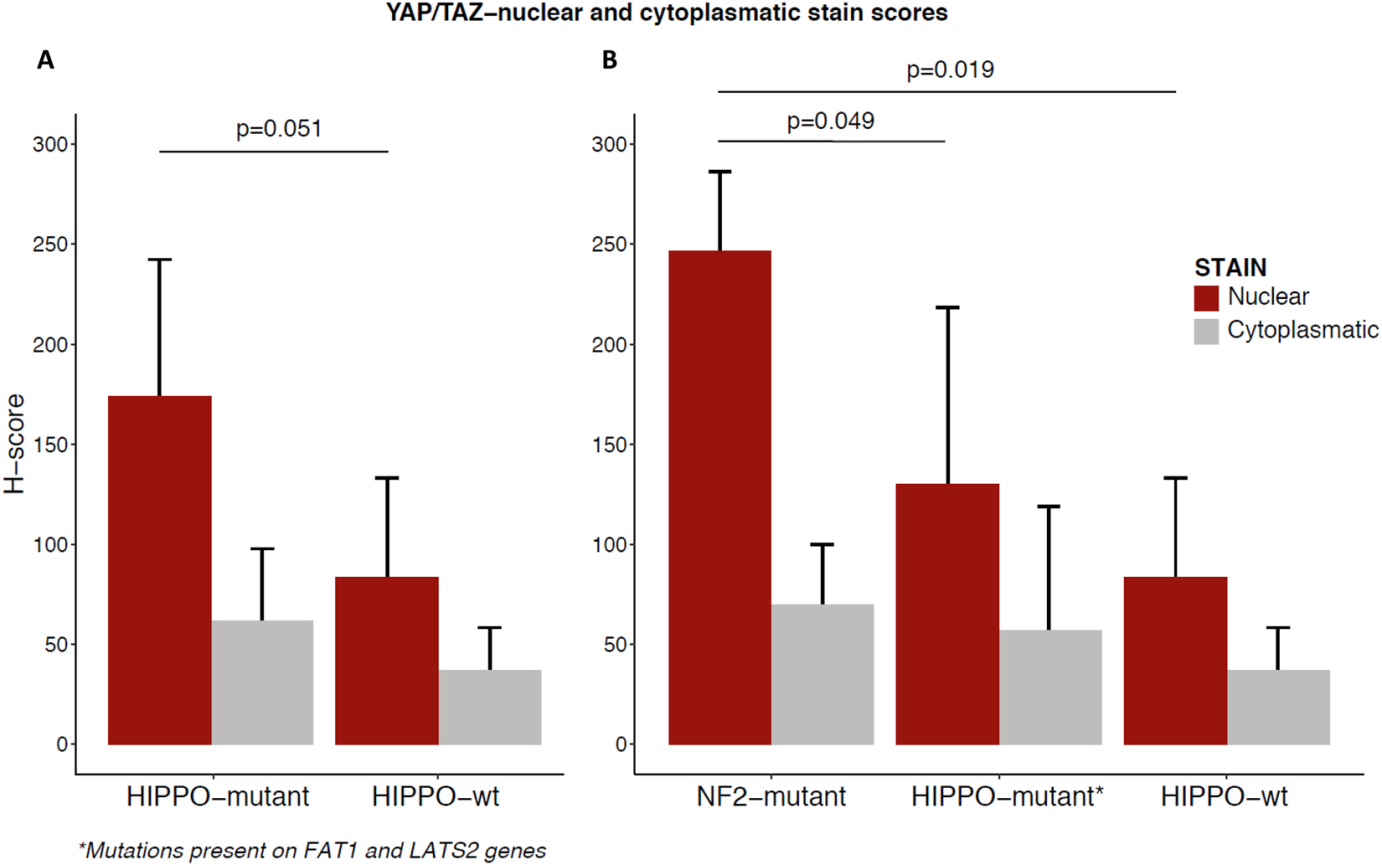
Immunohistochemistry staining for YAP/TAZ in sarcomatoid components of Hippo-mutant (N = 8) and wild-type (N = 8) sRCCs A, expression of YAP/TAZ in Hippo-mutant and Hippo wild-type sRCCs B, expression of YAP/TAZ according to Hippo-mutation type.

Several reports showed that *NF2* is a potent suppressor of hippo signalling through phosphorylation of YAP/TAZ leading to their sequestration in the cytoplasm and their degradation by the proteasome, thereby blocking oncogenic transcription associated with YAP/TAZ nuclear translocation^12^. To explore the relevance of mutations affecting Hippo genes in sRCC, we determined YAP/TAZ protein expression and intracellular localization by immunohistochemistry in 8 Hippo-mutated sRCC and 8 Wild-type sRCC. As expected, there was a trend toward increased nuclear YAP/TAZ signal in Hippo-mutant cases as compared to wild-type cases (p = 0.051) (Fig. 4A). Strikingly, when we focused on *NF2*-mutant cases (n = 3), we observed a stronger nuclear YAP/TAZ protein as compared to wild-type sRCC (p = 0.019) and other Hippo-mutants (*LATS1* and *FAT1*) (p = 0.049) (Fig. 4B), suggesting distinct functional importance of mutations affecting Hippo pathway components. Of note, no difference was observed between cytoplasmic YAP/TAZ signal between Hippo-mutant cases and Wild-type cases (p = 0.35).

### YAP1 knockout and NF2 reconstitution inhibit proliferation and invasion of sRCC

**Figure 5:**
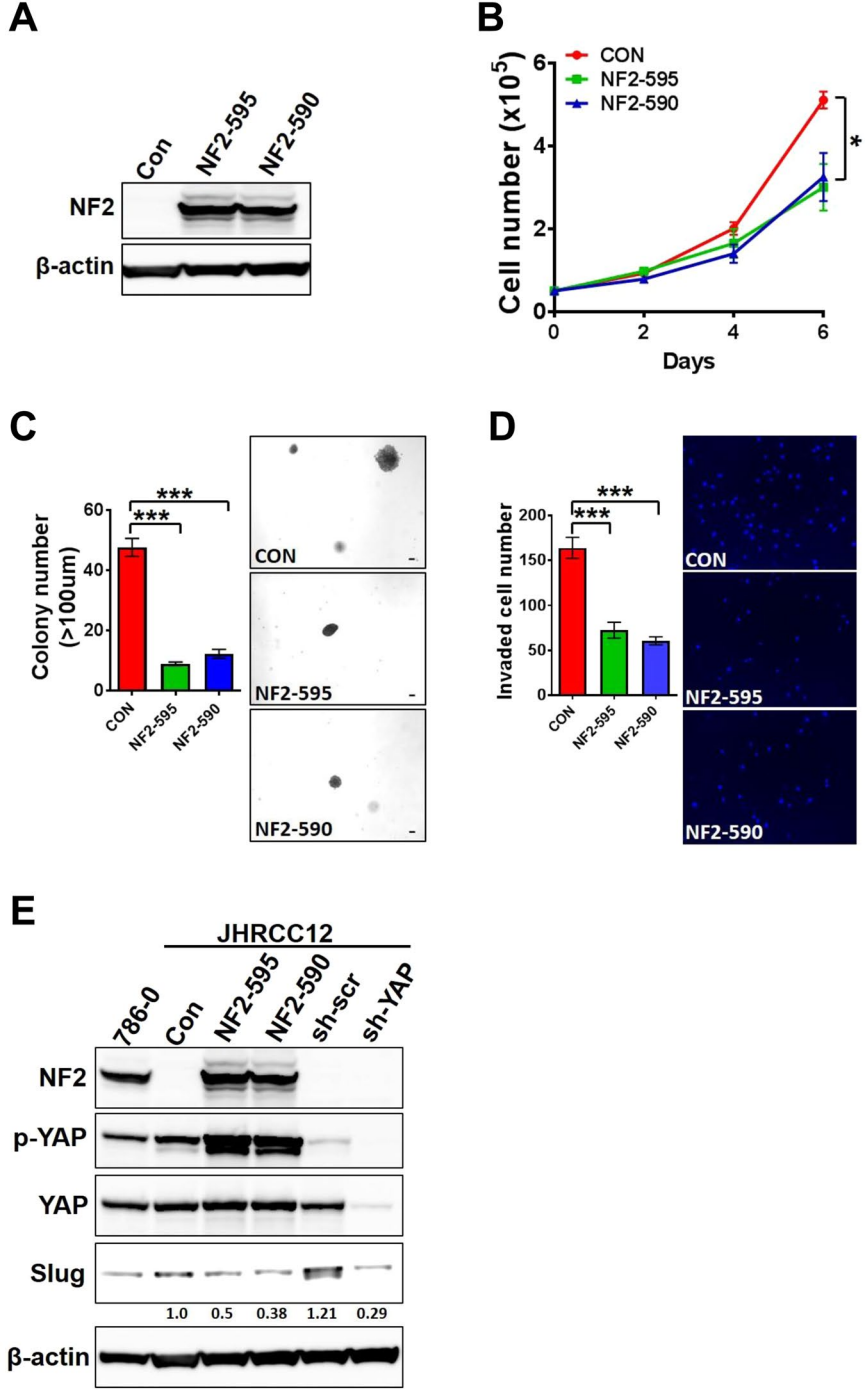
*NF2* reconstitution suppressed cell proliferation, transformation and invasion in *NF2*-mutant sRCC. (**A**) Immunoblot of *NF2* reconstitution expression in JHRCC12 cells. (**B**) Cell proliferation after *NF2* reconstitution was performed by cell counting on day 0, 2, 4, and 6. Data are presented as mean ± SD. *P < 0.05. (**C**) Soft agar assay for control and *NF2* reconstituted JHRCC12 cells. Scale bar is 100um. Data presented are mean ± SD. ***P < 0.001. (**D**) Invasion assay for control and *NF2* reconstituted JHRCC12 cells. Data presented are mean ± SD. ***P < 0.001. (**E**) Immunoblot of NF2, p-YAP, YAP, Slug and β-actin in 786-0, control, *NF2* reconstitution and *YAP1* knockdown JHRCC12 cells. Slug expression level was normalized to control expression sample.

**Figure 6:**
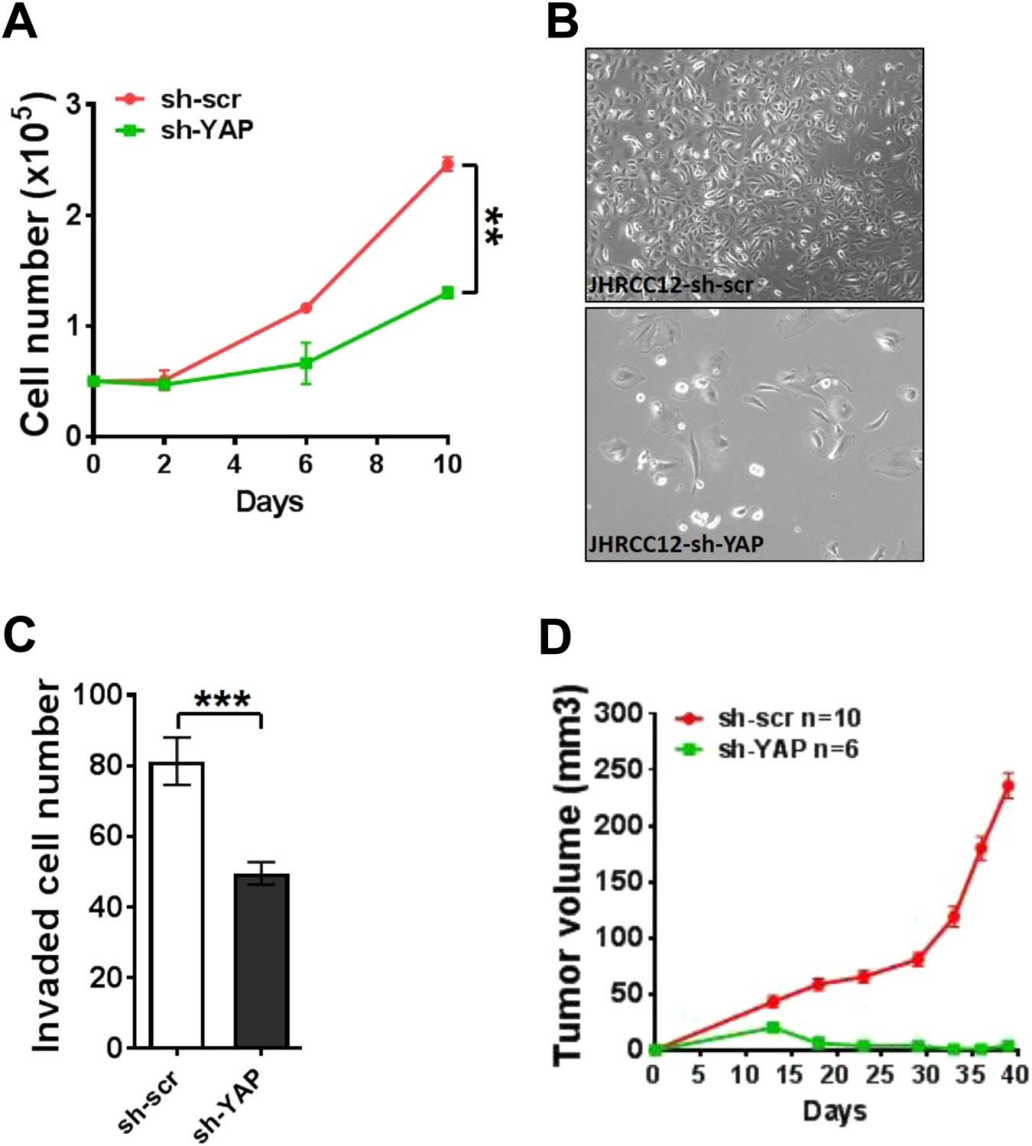
*YAP1* knockdown suppressed cell proliferation, invasion and tumour growth in *NF2*-mutant sRCC. (**A**) Cell proliferation of *YAP1* knockdown in JHRCC12 cells. Data are presented as mean ± SD. **P < 0.005. (**B**) Cell number and morphology change after YAP*1* knockdown. (**C**) Invasion assay for scramble-control (sh-scr) and *YAP1* knockdown (sh-YAP) in JHRCC12 cells. Data presented are mean ± SD. ***P < 0.001. (**D**) Tumour growth curve of scramble-control (sh-scr) and *YAP1* knockdown (sh-YAP) JHRCC12 cells.

To analyse the functional role of hippo core genes mutations, we used JHRCC12, a *NF2*-mutant sRCC cell line, as a model. Notably, *NF2* reconstitution (Fig. 5A) suppressed cell proliferation, transformation and invasion (Fig. 5B–D). As YAP1 expression has been shown to strongly induce expression of mesenchymal genes such as *SLUG* (*SNAI2*)^13^, we then investigated the effect of *NF2* reconstitution and *YAP1* knockdown on SLUG expression; as expected, we found decrease of SLUG which was more profound with *YAP1* knockdown than with *NF2* reconstitution (Fig. 5E). We then asked whether interference with hippo pathway could disrupt tumour growth *in vitro* and *in vivo*. *YAP1* knockdown in JHRCC12 cells suppressed cell proliferation, invasion, and tumour growth and induced change in their morphology (Fig. 6A–C). Finally, to explore the effect of YAP1 interference *in vivo*, we performed loss-of-function experiment in the male immunocompromised NOD-SCID IL2Rg−/− (NSG) mice using JHRCC12 xenografts. Notably, we observed that *YAP1* suppression blocked tumour growth (Fig. 6D), suggesting that proliferation *in vivo* occurs in cells with higher YAP1 activity. Together, the *in vitro* and *in vivo* observations suggest that the interference with Hippo pathway might have therapeutic relevance.

## Discussion

Our study highlights for the first time an association between alterations of the Hippo pathway, a key regulator of organ growth, and sarcomatoid dedifferentiation in ccRCC. Mutations of *NF2*, *FAT1* and *LATS2* were the most frequent Hippo pathway alterations^14^. Most notably, sRCCs exhibited more frequent mutations in Hippo genes than non-sRCC controls. We showed that downstream effectors of the Hippo pathway were upregulated in patients with alteration in Hippo pathway genes. Finally, mechanistic studies *in vitro* and *in vivo* demonstrated that *YAP1* inhibition or *NF2* reconstitution impaired tumour growth and proliferation. Thus, Hippo pathway mutations appear as one of the most frequent potential oncogenic mechanisms in sRCC. The finding of one tumour with deleterious *NF2* mutation in its sarcomatoid component only, suggest that Hippo pathway mutations might be late events in ccRCCs with sarcomatoid dedifferentiation.

The prevalence of Hippo pathway gene alterations in this study is supported by previous observations from Bi *et al*. of increased FAT family member mutations in the sarcomatoid component of kidney tumours^6^. However, various observations in our study contrast with previous molecular studies. Notably, the role of tumour suppressor *TP53* in sRCCs is still unclear, as we found a lower frequency of *TP53* alterations than expected, reported in 19% of sRCCs. Conversely, *TP53* mutations were found in 31% and 42%, respectively, of sarcomatoid kidney tumours with mixed histology in the studies by Bi *et al*.^6^ and Malouf *et al*.^4^. Such differences might be explained by the small numbers of patients, and heterogeneity of histologies in the previously published papers. Along with *TP53*, *TERT* was also recurrently altered in our sRCC cohorts, while such alterations were found in less than 3% of lower grade tumours datasets^15,16^. It is yet unknown whether this high rate of *TERT* and *TP53* alterations is related to subclonal evolution in aggressive and heterogeneous tumors, or could be directly associated with epithelial mesenchymal transition mechanisms. Thus, the importance of *TP53* and *TERT* in sRCC should be further explored in follow-up studies.

Our study also highlighted recurrent mutations of *NF2* in sRCCs, which contrasts with previous reports of *NF2* alterations that were specific to sarcomatoid tumours with papillary histology^5^. In addition, recent work suggested that the Hippo pathway is recurrently disrupted in aggressive, unclassified kidney tumours. In that study, loss of *NF2* and concurrent accumulation of *YAP*/*TAZ* in the nucleus occurred in 26 of 62 (26%) unclassified kidney cancers^17^. These data pinpoint that Hippo alterations might be a marker of tumour aggressiveness regardless of histology.

The putative role of the Hippo pathway in sarcomatoid dedifferentiation is supported by the promotion of epithelial-mesenchymal transition (EMT) by the core members of the Hippo pathway, *YAP1*^13,18,19^ and *TAZ*^20,21^. *YAP1* and *TAZ* are transcriptional regulators reported to be expressed upon TGF-β pathway activation, and are both associated with EMT signatures in solid tumours^13^ through upregulation of EMT genes vimentin, fibronectin, *SLUG* and *ZEB1*. An interplay between MAPK signalling and *YAP1* is also reported in various studies^13,19^. Our study confirm the importance of Hippo core pathway member YAP1, but also of upstream effector NF2 for the regulation of tumor morphology and invasive properties.

A comprehensive molecular characterization of 19 Hippo core genes was performed on 9,125 tumour samples across 33 cancer types from The Cancer Genome Atlas^22^. Among the hyper-altered cancer types, mesotheliomas and papillary renal cell carcinomas were the most significant, and this finding was likely related to a high frequency of *NF2*, *LATS2*, and *SAV1* mutations. Of note, YAP1/TAZ activation has been previously shown to confer malignant phenotypes to mesothelial cells and conditional (mesothelium-specific) *NF2* knockout mouse models exhibited an increased incidence of mesothelioma development^23^. Although function of the Hippo pathway in the kidney has been unknown for long time, recent studies have identified a key role of *YAP1* signalling in glomerular embryonic development, maintenance of podocyte homeostasis, and renal fibrogenesis^24^. Whether conditional (kidney-specific) Hippo knockout mouse models would be associated with kidney cancer development and/or sarcomatoid dedifferentiation remain unknown to last of our knowledge.

Targeting the Hippo pathway can be a valid approach in Hippo-driven malignancies. Currently, different approaches to block YAP1/TAZ interactions with their target transcription factors TEADs are explored. Verteporfin was the first small molecule to act as a YAP1-TEAD binding inhibitor; Other YAP1-TEAD inhibitors include the bioengineer-designer cyclic YAP-1 like peptides as well as the natural YAP1 antagonist VGLL4^12^. In a preclinical model of papillary renal cell carcinomas harbouring *NF2* loss^25^, inhibition of the YAP1 partner YES1 *by* dasatinib or saracatinib led to repression of Hippo transcriptional targets and provided potent antitumour activity. In addition, several distinct small molecules including dasatinib, pazopanib, statins, and ivermectin, have also been shown to inhibit YAP1/TAZ by drug screening in human cancer cell lines^26^. These data suggest that our findings can have immediate perspectives for clinical care.

Overall, our study identifies the Hippo pathway as a key alteration in sRCCs. These findings advocate for new therapeutic developments targeting the Hippo pathway in sarcomatoid renal cell carcinomas, and further investigation of this pathway’s oncogenic potential across other cancer subtypes.

## Methods

### Sample collection

Frozen or formalin-fixed, paraffin-embedded (FFPE) sRCC, tissue samples were collected from 49 patients who underwent nephrectomy in Memorial Sloan Kettering Cancer Center, NY, USA. All were de-identified. Dedicated genitourinary pathologists reviewed all samples to confirm the diagnosis of sRCC, defined as clear-cell RCC associated with any percentage of sarcomatoid component according to the International Society of Urological Pathology guidelines^27^. All patients had previously provided written informed consent for tumour collection and analysis.

We performed laser-assisted microdissection of samples of 27 tumours allowing targeted sequencing of the epithelial and mesenchymal components. We expanded that cohort with 22 primary sRCC samples that did not undergo microdissection. Local controls comprised non-sRCC samples from 268 patients.

The Memorial Sloan Kettering Cancer Center’s Institutional Review Board approved the study, and the collection and use of tissues followed ethical procedures formulated in the Helsinki Declaration

### Targeted sequencing

We performed targeted sequencing using the MSK-IMPACT assay^28^. Samples from the 27 patients were analysed on a panel of 341 key cancer-associated genes, and samples from the other 22 patients were analysed by an updated assay that includes 410 genes. Full lists of the genes in both assays are included as Supplementary Table S3. Barcoded sequences were prepared and captured by hybridization with custom biotinylated DNA probes for all exons and selected introns of the selected genes using 100–500 ng of input DNA. Captured libraries were sequenced on an Illumina HiSeq (2 × 100 bp paired-end reads). The raw reads were aligned to the human genome (hg19) using the Burrows-Wheeler Alignment Tool, followed by duplicate read removal, base recalibration and indel realignment using GATK (v 2.6–5)57. Somatic variants were called using MuTect (v 1.1.4) for single-nucleotide variants and Somatic Indel Detector (GATK 2.3–9) for indels; they were then annotated by Annovar for cDNA and amino-acid changes as well as their presence in the dbSNP database (v137) and COSMIC database (v68) and in the 1000 Genomes database for minor allele frequencies. IMPACT was designed to focus on somatic mutation detection by filtering out alterations also present in normal samples. We assessed the functional impact of single nucleotide variants using 2 independent web-based tools, Mutation Assessor^29^ and Polyphen2^30^.

### Statistical analysis

Mapping of the Hippo protein alterations was performed using MutationMapper v1.0.1^31,32^. We associated mutated genes with oncogenic pathways according to comprehensive analysis of the published literature^14,33–36^. Comparisons of clinical, pathological and molecular data were performed with the Fisher exact test or Wilcoxon test, depending on the study variable. All statistical tests were 2-sided and conducted at the significance level of 0.05.

### Animal studies

All animal work was performed in accordance with institutional guidelines and Institutional Animal Care and Use Committee (IACUC) approval. Five-to-six-week-old male immunocompromised NOD-SCID IL2Rg−/− (NSG) mice (Jackson Laboratory) were used for the study. To generate xenograft tumour growth curve, one million cells resuspended in PBS:matrigel (1:1) were injected into the flank of the mice subcutaneously. Two flank tumours were placed per mouse. Tumour volume was measured using calipers (calculated as 0.5 × L xW2), and growth curves were generated using GraphPad Prism software.

### Plasmid constructs and virus production

Full-length NF2 isoforms 1 (amino acid 1–595) and 2 (amino acid 1–590) were digested and cloned into pBabe-puro plasmid at the BamHI and EcoRI sites. pLKO1-shYAP plasmid (addgene 27369) was used for *YAP1* knockdown. Both retrovirus for *NF2* reconstitution and lentivirus for *YAP1* knockdown were generated by transfecting HEK293T cells with Lipofectamine 2000 reagent (Invitrogen). Cell culture medium with virus was collected and centrifuged at 3000 g for 5 minutes at 4 °C. The supernatant was used for target cell infection with 8ug/ml polybrene. The infected cells were selected in 2 ug/ml puromycin in culture medium and used for assays.

### Cell culture, proliferation and western blot

Cell culture was performed as describe before^37^. For cell proliferation assay, 5 × 104 cells were seeded in a 6-cm dishes in triplicate on day0 and counted on different time points indicated in the figures by cell counter (Nexcelom, USA). Cell proliferation curves were generated with GraphPad Prism software v.5.02 (La Jolla, CA, USA). For western blot, cells were lysed in standard RIPA buffer. Antibodies for NF2 (abcam ab88957), p-YAP (Cell Signaling 13008), YAP (Cell Signaling 12395), Slug (Cell Signaling 9585) and β-actin (Sigma A1978) were used for western blot.

### Soft agar assay and invasion assay

For soft agar assay, 1 × 105 cells were seeded onto a 6-cm dish containing a top layer of 0.3% noble agar and a bottom layer of 0.6% noble agar base. Cells were fed with media every 3 days. After 3 weeks, colonies with diameter larger than 100 μm were scored. Three independent triplicate experiments were performed. Scale bar is 100um. Data presented are mean ± SD of three independent experiments.

For invasion assay, 2 × 104 cells seeded in each transwell of 24-well (8um) Corning invasion chamber in culture medium with 0.1% FBS. Full culture medium with 10% FBS was added in each well of the plate as the attractant. After 24-hour incubation at 37 °C, cells in each transwell were removed and the membrane was fixed in 10% formalin and stained with DAPI solution. Invaded cell number was counted in 9 random views from triplicate under fluorescence microscope.

### Immunohistochemistry

Immunohistochemistry was conducted in 5 µm formalin-fixed paraffin-embedded (FFPE) whole tissue sections. Staining for YAP/TAZ (D24E4, Cell Signalling Technology, 1:50) was performed using an automated Ventana Discovery system (Ventana). Immunostaining scores (H-scores) for YAP/TAZ nuclear and cytoplasmic staining in the sarcomatoid areas were assessed separately and calculated as [H = intensity (0-3) × percentage of positive cells (1–100)].

## Supplementary information

Supplementary Dataset 1.

Supplementary Dataset 2.

Supplementary Dataset 3.
